# The Long Run towards Personalized Therapy in Non-Small-Cell Lung Cancer: Current State and Future Directions

**DOI:** 10.3390/ijms24098212

**Published:** 2023-05-04

**Authors:** Carlo Genova

**Affiliations:** 1 Dipartimento di Medicina Interna e Specialità Mediche, Università degli Studi di Genova, 16132 Genova, Italy; carlo.genova@hsanmartino.it; 2 UO Clinica di Oncologia Medica, IRCCS Ospedale Policlinico San Martino, 16132 Genova, Italy

Non-small-cell lung cancer (NSCLC) is the major cause of cancer-related deaths worldwide, due to its high incidence and mortality [1]. The challenge posed by this disease has led to an impressive scientific development in terms of knowledge of molecular pathways associated with tumor progression and innovative therapeutic approaches; hence, the current therapeutic landscape of NSCLC is notably complex and strictly linked to the molecular profile of each specific tumor. This concept is extremely relevant, as it implies that the evolution of NSCLC management has proceeded in parallel with the growth of molecular biology and the affirmation of approaches based on precision medicine [2,3]. Indeed, the optimal treatment of advanced NSCLC requires the evaluation of multiple actionable oncogenic alterations in genes involved in cell proliferation. Notably, physicians involved in thoracic malignancies have become used to the periodic update of therapeutic algorithms due to the periodic inclusion of novel oncogenic drivers, and nowadays, approximately 15–20% of newly diagnosed NSCLC patients harbor an actionable oncogenic driver [4]. While most patients with advanced NSCLC do not harbor actionable drivers (as far as we know) and are hence eligible for treatment with immune checkpoint inhibitors, either alone or in combination with chemotherapy, appropriate molecular characterization still plays a role in this patient subgroup. Indeed, on the one hand, it is acknowledged that most major oncogenic drivers (such as *EGFR* and *ALK*) are associated with poor response to immunotherapy, and failing to identify such patients results in poor outcomes [5,6]; on the other hand, the emerging mutations not considered drivers but with potential impact on the efficacy of immune checkpoint inhibitors, such as *KEAP1* or *STK11*, as well as other molecules involved in tumor microenvironment, are becoming a subject of interest in immunotherapy research, and might eventually reach relevance for introduction in clinical practice [7,8]. Previously, the relevance of molecular characterization for NSCLC has been limited to the advanced stage; however, the role of targeted therapies in localized NSCLC are being explored in clinical trials, some of which are currently ongoing [9,10]. The most prominent example of this evolution is the ADAURA study, which demonstrated outcome improvements with adjuvant osimertinib [11].

The first consideration based on the current context is that the technology for the detection of molecular alterations is rapidly and constantly improving, with increasing sensitivity in terms of gene detection, including both acknowledged and novel, uncommon mutations; the second consideration takes into account the increasing availability of targeted therapies, with a non-negligible degree of variation among the different regulatory agencies. These improvements pose further challenges: on the one hand, pathologists and molecular biologists must continuously make efforts in terms of training and upgrades of laboratory tools; on the other hand, medical oncologists are required to make clinical decisions for patients with uncommon molecular alterations (including uncommon mutations in both acknowledged targets and uncommon novel targets), for which limited research is available so far, and targeted agents may exist while not being readily available through national healthcare services. An emerging approach to address these potential issues is represented by the institution of molecular tumor boards, which are expected to include personnel with the skills and resources needed for the optimal management of patients whose tumor harbors a complex molecular profile [12].

Another concept, strictly linked with the development of molecular biology and targeted therapy, is represented by the study of resistance mechanisms developed during treatment with targeted agents, which result in clinical disease progression. Notably, the identification of said mechanisms is expected to result in the development of novel therapeutic strategies designed to either manage and counteract resistance-based progression, or to prevent the occurrence of acquired resistance in first place [13]. While the latter approach seems reasonable to prevent the most common acknowledged mechanisms of resistance, such as the development of *MET* alterations during treatment with *EGFR* inhibitors, the former approach appears more suitable when various, singularly uncommon, acquired mutations occur. One major limit to this approach is the necessity to re-characterize the tumor at progression, with potential discomfort for the patient and perception of time loss, especially when new actionable drivers are not identified. One possible answer to this issue is represented by the employment of liquid biopsy to detect novel mutations from peripheral blood. This emerging approach is rapidly evolving in terms of accuracy, and its potential applications are currently being explored in the translational and clinical setting. In particular, by making the molecular characterization more accessible compared with conventional biopsy, blood-based liquid biopsy allows for repeated sample collection from each patient, thus providing an unprecedented opportunity for longitudinal monitoring. Furthermore, the nature of liquid biopsy should make its molecular profile representative of the whole neoplasm, hence overcoming the limits of conventional biopsy due to tumor heterogeneity. Notably, while liquid biopsy is extremely promising, histology-based assessments, such as histotype diagnosis and the identification of morphologic shift during targeted therapy (including transformation to small-cell lung cancer) are still beyond the possibilities of liquid biopsy [14,15,16].

Similar to other fields of medicine, molecular oncology is expected to benefit from the increasing applications of artificial intelligence (AI). In this context, predictive algorithms can provide a large amount of information able to aid in histomolecular diagnosis; two major examples of these applications are represented by digital pathology and radiomics. In digital pathology, AI-based algorithms are applied to scanned tumor slides in order to detect features invisible to the human eye and predictive of specific molecular profiles [17]. In radiomics, a similar concept is applied to radiologic imaging, such as computed tomography scans, in order to detect features associated with an increased probability of harboring particular molecular profiles, such as activating mutations of *EGFR* [18]. The development of such technologies might eventually lead to wide use in common practice, with subsequent improvements in personalized medicine.

In conclusion, molecular oncology and precision medicine have made a huge leap in the last few years, resulting in significant survival advantages in different malignancies, and particularly in NSCLC. However, in spite of the impressive achievements, there is still huge room for improvement. The continuous study of emerging molecular alterations and the development of novel treatments capable of exploiting oncogenic drivers remain major objective in thoracic oncology.
